# The Effects of Varying Concentrations of Didecyl Methylpropyl Ammonium Iodide (DMPAI) on the Structure and Function of Soil Bacterial Communities in the Lake–Terrestrial Ecotone

**DOI:** 10.3390/microorganisms13040934

**Published:** 2025-04-18

**Authors:** Qi Zhu, Lingquan Zeng, Chunhua Li, Chun Ye

**Affiliations:** National Engineering Laboratory for Lake Pollution Control and Ecological Restoration, State Environmental Protection Key Laboratory for Lake Pollution Control, Chinese Research Academy of Environmental Sciences, Beijing 100012, China; zhu.qi@craes.org.cn (Q.Z.); cenglingquan22@mails.ucas.ac.cn (L.Z.)

**Keywords:** lake–terrestrial ecotone, bacterium, algaecide, QACs, gene function

## Abstract

To address freshwater lake blooms resulting from eutrophication, the application of quaternary ammonium compounds as algaecides serves as an effective emergency remediation strategy. Didecyl methylpropyl ammonium iodide (DMPAI) is a novel quaternary ammonium algaecide; however, its bacteriostatic properties may significantly disrupt the microbial activity in lakes, particularly within the lake–terrestrial ecotone. To investigate the degradation process of DMPAI in the lake–terrestrial ecotone and its impact on the microbial community, experiments were conducted using a large-scale indoor simulation device to analyze DMPAI concentrations, the composition of the lake microbial community, and associated gene functions. The results showed that (1) DMPAI was completely removed from the lake water body in approximately 36 h; (2) The addition of DMPAI altered the microbial community structure in the lake–terrestrial ecotone, as evidenced by an increase in the diversity index and the proliferation of microorganisms capable of tolerating and degrading DMPAI, such as *Pseudomonas* and *Flavobacterium*, within a short period. These changes were typically observed after 10 d and generally recovered, not persisting for extended periods. (3) Functional genes involved in carbon, nitrogen, and sulfur cycling are more significantly impaired in the lake–terrestrial ecotone with DMPAI addition. The destabilization of the microbial community may lead to a short-term increase in pathogenic bacteria during the recovery process. This phenomenon was more pronounced in environments with higher concentrations of DMPAI. Therefore, the concentration of DMPAI should be controlled within the range of 0.5 to 2.0 mg L^−1^.

## 1. Introduction

The rapid development of human society has led to significant volumes of wastewater, rich in nitrogen and phosphorus, being discharged directly or indirectly into lake ecosystems, contributing to eutrophication and the resultant water blooms, which have become among the most pressing environmental issues affecting freshwater lakes [1]. Since the 1980s, algal blooms have been documented in approximately 68% of lakes worldwide [1]. Severe algal blooms have been observed in three of China’s great lakes—Dianchi Lake [2], Taihu Lake [3], and Chaohu Lake [4]—while cyanobacterial blooms were also reported in Wuhan’s Guanqiao Lake and Shuiguo Lake during 2009 and 2010. Algal blooms typically produce a range of toxic substances, with microcystins (MCs) being among the most commonly reported and posing a significant threat to human health [5]. Taihu Lake and the Yangtze River in China have been experiencing water bloom issues for over 20 years, with MCs in the Taihu Lake and Chaohu Lake regions in 2015 exceeding the relevant Chinese standards by a factor of 2600 [6,7,8]. The Great Lakes basin in the United States has also faced severe algal bloom events and livestock fatalities, with the dominant toxin-producing alga being *Microcystis* spp., which produces MCs [9]. Therefore, managing the water bloom crisis resulting from the eutrophication of water bodies has become a critical issue of significant concern in the field of lake ecology and environmental management, both in China and globally.

Currently, methods for controlling *Microcystis* blooms are primarily classified into the following three categories: physical, biological, and chemical methods. Among these, the chemical algaecide method is typically employed to address emergency bloom outbreaks [10]. This method offers the advantages of a low dosage and rapid effect, but it can lead to secondary pollution and pose ecological risks to the water body. Quaternary ammonium compounds (QACs) are a class of cationic surfactants. As algaecides, QACs offer advantages such as good stability, low toxicity, non-irritating properties, reliable action, and the ability to effectively remove phytoplankton from water under alkaline conditions [11]. In recent years, numerous scholars have suggested using QACs for managing bloom algae, such as *Microcystis aeruginosa*, with promising results. However, QACs also exert strong inhibitory effects on environmental microorganisms. QACs primarily alter the phospholipid bilayer of the cell membrane via the alkyl chain, disrupting the membrane and causing the loss of intracellular substances, ultimately leading to microorganism death [12]. Residues of quaternary ammonium compounds in the environment have been found to potentially disrupt ecosystem functions by altering microbial community structures. This ecological risk is particularly pronounced in the lake–terrestrial ecotone, which has unique ecological functions.

DMPAI is a quaternary ammonium compound (QAC) and an emerging algaecide that has been extensively applied in China to manage algal blooms in freshwater bodies. It has the chemical formula C_24_H_52_IN and features a chain-like molecular structure with active branches. In comparison with other QACs such as benzalkonium chloride (BAC), DMPAI demonstrates lower toxicity to zooplankton and aquatic vertebrates [13]. Exposure to DMPAI concentrations below 2 mg/L showed no observable impact on the normal physiological behavior of Daphnia magna and Danio rerio [14]. Additionally, DMPAI exhibits notable antibacterial activity. Research indicates that DMPAI at concentrations between 1 and 5 mg/L is capable of significantly suppressing the growth of multiple pathogens, including *Staphylococcus*, *Edwardsiella*, *Vibrio*, and *Bacillus*.

The lake–terrestrial ecotone, as a transitional zone between land and water, serves as both a natural barrier for intercepting pollutants and a critical interface for biogeochemical cycles. Additionally, the soil microbial community in the lake–terrestrial ecotone plays a central role in maintaining the stability of the wetland ecosystem. Studies have shown [15] that microbial communities are crucial for organic matter decomposition, nitrogen and phosphorus cycling, and pollutant degradation in the lake–terrestrial ecotone. Soil microbial communities are highly sensitive to chemical disturbances, and exposure to quaternary ammonium salts at concentrations as low as 0.1 mg L^−1^ can significantly reduce the abundance of nitrifying bacteria. However, most current studies focus on the effects of quaternary ammonium salts on microorganisms in aquatic environments [16,17], leaving a significant research gap regarding this particular habitat in the lake–terrestrial ecotone. Notably, DMPAI, as a QAC, possesses a long alkyl chain structure, which may enhance its adsorption to soil organic matter, posing a risk of persistent pollution. Therefore, the effect of DMPAI on the structure and function of soil microbial communities in the lake–terrestrial ecotone is a critical issue that requires urgent study.

In this study, we created a near-natural lake–terrestrial ecotone environment using a large-scale indoor simulation device, coupled with 16S rRNA high-throughput sequencing, to investigate the degradation rate of DMPAI in the lake–terrestrial ecotone and the short-term effects of various concentrations of DMPAI on the structure of the soil bacterial community and functional genes. This study was predicated on the following three core hypotheses: (a) rapid biodegradation would occur for DMPAI in lake–terrestrial ecotones; (b) bacteriostatic effects would induce transient, recoverable disturbances in microbial community structure; and (c) biogeochemical cycling functions would exhibit concentration-dependent impairment. The results of this study will contribute to further assessing the environmental risks of DMPAI and optimizing its concentration as an algaecide, which is crucial for enhancing the ecological protection of the lake–terrestrial ecotone and ensuring ecological safety in cyanobacterial management.

## 2. Materials and Methods

### 2.1. Experimental Materials

**Figure 1:**
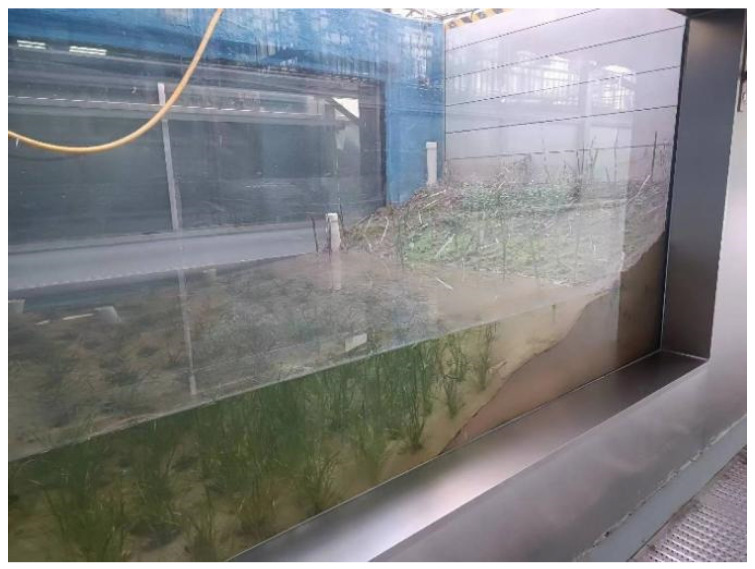
Schematic diagram of the indoor ecological simulation device for the lake–terrestrial ecotone.

The study was conducted using an indoor lake–terrestrial ecotone ecological simulation device at the National Engineering Laboratory for Lake Pollution Control and Ecological Restoration, Chinese Research Academy of Environmental Sciences (Figure 1). The effective dimensions of the lake–terrestrial ecotone ecological simulation pools were 3.0 m in length, 2.1 m in width, and 2.0 m in height. Acrylic observation panels (2.82 m × 1.62 m) were installed on both sides of the simulation pool. To ensure waterproofing and corrosion protection, the interior of the pool was coated with blue epoxy. The device was capable of fully simulating the environmental conditions of a natural lake by adjusting the water level, water quality, light intensity, and even simulating rainfall through a computerized control system. The thickness of the sediment layer in the simulation pool was maintained at 15 cm, while the water layer thickness was maintained at 50 cm. A constant temperature of 25 °C and a photoperiod of 12 h per day were maintained in the simulation pool. The pool was regularly supplemented every 30 days using an artificial rainfall system operating at a rate of 30 mm/h. The chemical properties of the soil used in the simulation pool are detailed in Table 1.

### 2.2. Experimental Methods

About 10 L of water was collected from the simulation pool in the lake–terrestrial ecotone and placed in a polypropylene incubation bucket (Nalgene, Rochester, NY, USA) with a capacity of 20 L. The water solution was thoroughly mixed with DMPAI solution to achieve a concentration of 2.0 mg L^−1^. Following the initiation of the experiment, water samples were collected every 4 h, and the concentration of DMPAI was measured to analyze its degradation in the lake–terrestrial ecotone water body. The experiment was conducted at 25 °C, with three parallel samples for each treatment.

Additionally, algaecide solutions with concentration gradients of 0.01 mg L^−1^ (treatment A), 0.5 mg L^−1^ (treatment B), and 2.0 mg L^−1^ (treatment C) were prepared. A total of 0.01 mg L^−1^ is generally considered to be the lowest concentration capable of affecting microorganisms. A total of 0.5 mg L^−1^ and 2.0 mg L^−1^ are commonly used concentrations for DMPAI as an algaecide [14]. A 300 mL conical flask was filled with 200 mL of DMPAI solution at different concentrations and 50 g of soil from the indoor lake–terrestrial ecotone (Figure 1), to simulate the lake–terrestrial ecotone environment. Bacteria in the sediments were utilized as research subjects to analyze changes in the composition of bacterial communities in the lake–terrestrial ecotone sediments using 16S rRNA high-throughput sequencing on days 0, 3, 6, and 10 of the experiment. The sample collected on day 0 served as the control. The soil samples collected were numbered sequentially from A1, B1, and C1 to A4, B4, and C4.

#### 2.2.1. Determination of DMPAI Concentration

A volume of 200–400 mL of the water sample was accurately pipetted into a dispensing funnel. Subsequently, 0.1 mol/L aqueous sodium thiosulfate solution was added, followed by 10 mL of ethylene dichloride and bromophenol blue indicator. The substance to be measured was extracted into the organic phase layer with thorough shaking and allowed to partition. The lower organic phase was then collected, and the concentration of DMPAI was precisely determined using a UV spectrophotometer, with dichloroethane used as the blank [18].

#### 2.2.2. 16S rRNA Sequencing

Total community genomic DNA extraction was performed using the E.Z.N.A. soil DNA kit (Omega, M5635-02, Stamford, CT, USA), following the manufacturer’s instructions. The DNA concentration was measured using a Qubit 4.0 (Thermo, Waltham, MA, USA) to ensure the extraction of adequate amounts of high-quality genomic DNA.

#### 2.2.3. Function Prediction

The cycling functions of soil bacteria, including carbon, nitrogen, and sulfur, were predicted using FAPROTAX software(version 1.2.1). Additionally, bacterial functional prediction analysis was conducted using PICRUSt (v1.1.4).

## 3. Results

### 3.1. Degradation Characteristics of DMPAI in the Lake–Terrestrial Ecotone Waters

**Figure 2:**
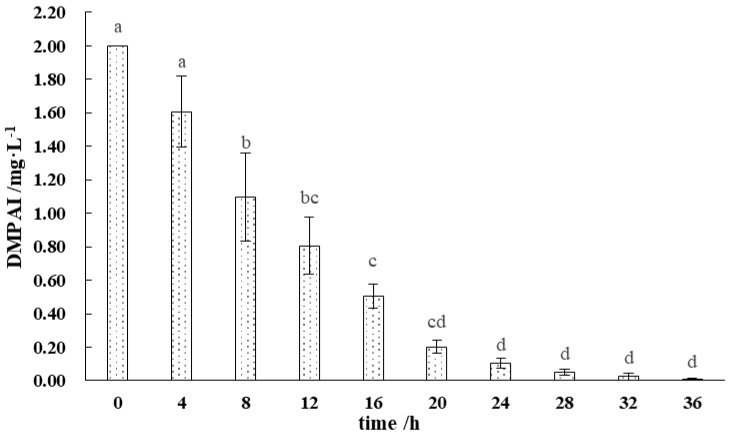
Distribution pattern of DMPAI degradation in lake−terrestrial ecotone waters. (Different letters indicate significant differences between treatment groups).

The degradation of DMPAI in the lake–terrestrial ecotone water body is illustrated in Figure 2. The period from 0 to 24 h after the addition of DMPAI was critical for its degradation, during which more than 95% of DMPAI was degraded. The highest slope of the curve was observed between 4 and 8 h, indicating that the degradation rate of DMPAI peaked. After 20 h, the degradation rate of DMPAI slowed significantly (*p* < 0.01). After 36 h, DMPAI was completely removed from the water column. In the lake–terrestrial ecotone water–soil system, the concentration of DMPAI decreased more rapidly. The concentration of DMPAI in the water is usually no longer detectable after 12 h. This is due to the fact that DMPAI is first adsorbed on the surface of the soil particles and then decomposed by microorganisms. These results suggest that soil microorganisms are the primary drivers of DMPAI degradation. DMPAI is typically adsorbed onto the surface of soil particles and subsequently decomposed by microorganisms. The retention time of DMPAI as an algaecide in the lake–terrestrial ecotone is relatively short, and it does not cause long-term stress to environmental microorganisms.

### 3.2. Response of Soil Bacteria to DMPAI in the Lake–Terrestrial Ecotone

#### 3.2.1. Effect of DMPAI on Bacterial Community Species Diversity

**Figure 3:**
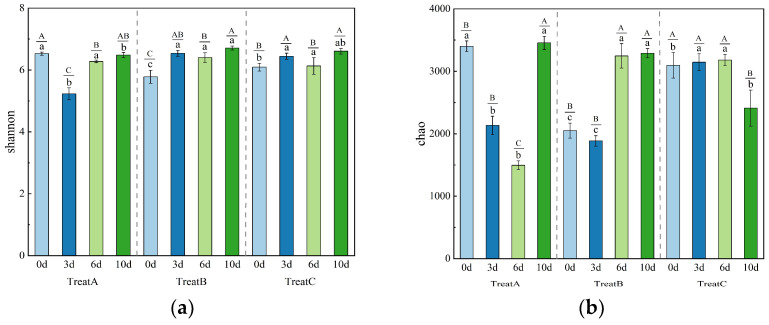
Histogram of change in Chao (**a**) index and Shannon (**b**) index of soil bacteria under different concentrations of DMPAI treatments. (Superscript Capital letters (A, B, C) indicate significant differences among time points within the same treatment, while lowercase letters (a, b, c) indicate significant differences among treatments at the same time point. Different letters denote statistically significant differences between groups (*p* < 0.05), whereas the same letter indicates no significant difference).

As shown in Figure 3, the effects of different DMPAI concentrations on the microbial diversity index in the soil varied. The Shannon index reflects the degree of uncertainty within microbial communities, providing a measure of both abundance and evenness, and serves as a key indicator of microbial community diversity. In the three treatments with varying DMPAI concentrations, the Shannon index initially decreased, followed by an increase from 0 to 10 d. By the end of the experiment, the Shannon indices for all treatments were comparable, ranging from 6.48 to 6.71. The experimental results indicated that the most significant changes in the Shannon indices of soil bacterial communities occurred primarily between 0 and 6 d following the addition of DMPAI. Although the trend of the Shannon indices varied across treatments, they consistently returned to a similar level after 10 d. The Shannon index results from the three treatments revealed a decrease followed by an increase in microbial community diversity.

The Chao index serves as an indicator of microbial species population size in the environment. The Chao index exhibited considerable variation under the influence of different DMPAI concentrations. In the case of low DMPAI concentration (treatment A), a continuous decrease in the Chao index of soil bacteria was observed from 3401.57 to 1495.00 within 6 d. During the period from 6 to 10 d, the Chao index increased rapidly to 3455.49, reflecting a 131.14% rise. The trend of the Chao index in treatment B closely resembled that in treatment A. In the high-concentration treatment (C), the Chao index remained within the range of 3095.90 to 3181.29 from 0 to 6 d, before sharply decreasing to 2410.70 on the day 10. These results indicate that DMPAI concentrations lower than 0.5 mg L^−1^ caused a dramatic decrease in the number of soil bacterial species within a short period. In contrast, the effect of 2.0 mg L^−1^ DMPAI on the number of soil bacterial species was delayed.

In conclusion, increasing the concentration of added DMPAI had a more significant positive effect on the Shannon index of soil bacteria by the end of the experiment. This phenomenon may be attributed to DMPAI disturbing the structure of the soil bacterial community, leading to the extinction of the original dominant species. Consequently, it promoted the growth of a variety of new species, thereby enhancing the homogeneity of the bacterial community. The low concentration of DMPAI drastically reduced the bacterial species in the soil within 3 to 6 d, but they largely recovered after 10 d without causing long-term effects on the bacterial community. The addition of DMPAI at a concentration of 0.5 mg L^−1^ substantially increased the number of bacterial species in the soil of the lake–terrestrial ecotone. Further increasing the concentration of DMPAI from this point may lead to a reduction in the number of soil bacterial species. Unlike the low concentration, this change occurred after 6 to 10 d. The concentration of DMPAI was not high enough to cause a decrease in the number of bacterial species. Therefore, controlling the concentration of DMPAI (<2.0 mg L^−1^) can effectively mitigate its negative effects on bacteria in the environment.

#### 3.2.2. Soil Bacterial Community Evolution in the Lake–Terrestrial Ecotone

**Figure 4:**
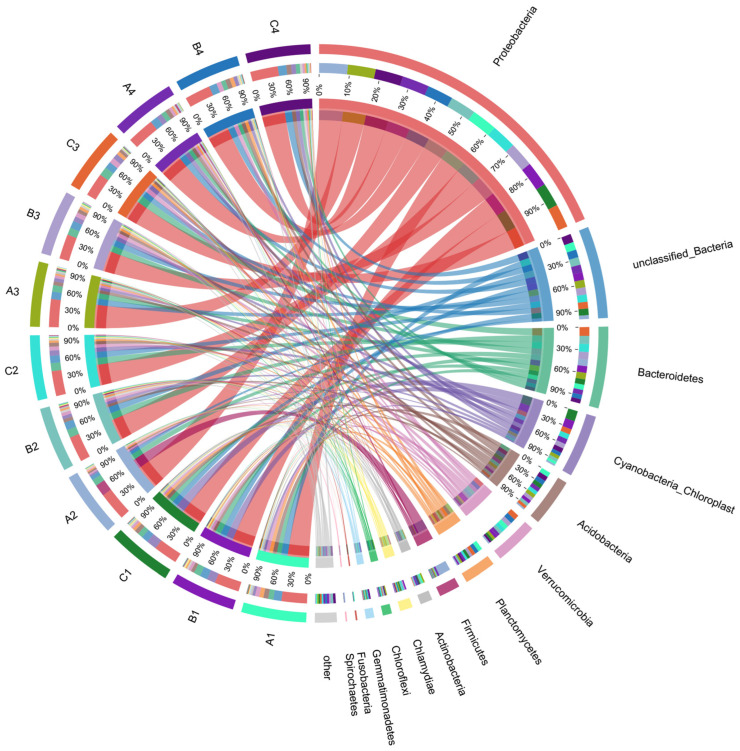
The covariance distribution of major phylum in the bacterial communities across different treatments in the experiment.

After performing 16S rRNA high-throughput sequencing analysis, the distribution of major soil bacterial phyla across treatments is presented in Figure 4. The results indicate that *Proteobacteria* was the co-dominant phylum across the various treatment groups, with relative abundance ranging from 38.77% to 47.03%. The relative abundance of *Proteobacteria* slightly increased in all treatments following the addition of varying concentrations of DMPAI. *Proteobacteria* encompass a wide range of organic pollutant-tolerant and degrading bacteria, which allow them to gain a competitive advantage in polluted environments.

Additionally, the application of DMPAI had a significant effect on the relative abundance of other dominant phyla within the community. The relative abundance of *Bacteroidetes* in the lake–terrestrial ecotone soils ranked second only to that of *Proteobacteria*. *Bacteroidetes* possess a phosphorus-solubilizing effect in soils and can convert difficult-to-degrade inorganic and organic phosphorus compounds into more accessible phosphorus forms [19]. Additionally, *Bacteroidetes* play a significant role in the degradation of organic matter [20]. After the addition of DMPAI for 10 d, the relative abundance of *Bacteroidetes* in the treatment group decreased from 8.68~10.92% to 6.80~8.03%. *Verrucomicrobia* plays a crucial role in the degradation of organic matter [21] and is widely distributed in various soil and freshwater environments [22]. The relative abundance of *Verrucomicrobia* is affected differently by varying concentrations of DMPAI. In treatment A, the relative abundance of *Verrucomicrobia* first decreased, then increased, while the opposite trend was observed in treatment C. *Acidobacteria* are an important group of soil bacteria associated with the degradation of phosphorus and complex organic matter [23]. In soil, they degrade plant residues and participate in the material cycle and ecosystem-building processes, such as iron cycling, metabolism of monocarbon compounds, and photosynthesis [24]. The relative abundance of *Acidobacteria* in all treatments decreased from 0 to 3 d at the beginning of the experiment, but by the end of the experiment (10 d) it recovered to, or even exceeded, the initial level.

**Figure 5:**
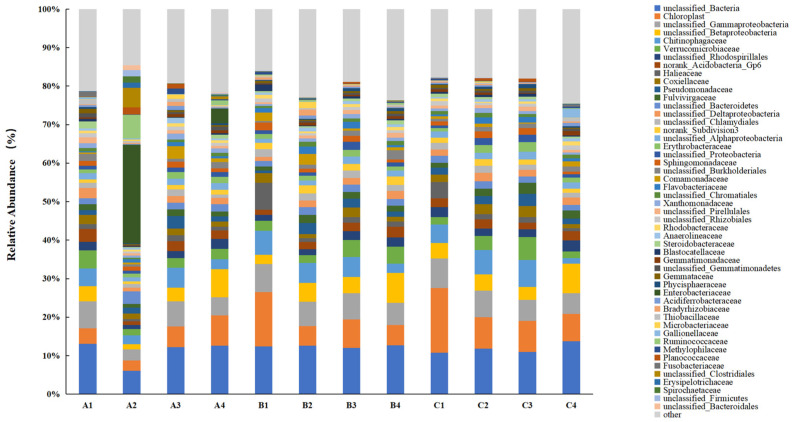
Distribution histogram of the main families of soil bacteria in the lake–terrestrial ecotone.

At the family level classification of the bacterial community (Figure 5), unclassified *Betaproteobacteria*, *Chitinophagaceae*, and *Verrucomicrobiaceae* were the predominant families. After the addition of DMPAI, the relative abundance of *Betaproteobacteria* in all three treatment groups increased substantially by the end of the experiment, with the average relative abundance rising 120.01% compared to 0 d. Studies have shown that *Betaproteobacteria* are characterized by nutrient-philic traits [25]. The continuous enrichment of nutrient substrates due to the application of algaecide may have contributed to the increased relative abundance of *Betaproteobacteria* in the soil. Additionally, after 9 d of the experiment, the relative abundance of *Chitinophagaceae* decreased by 58.46% from the initial value at 0 d. Previous studies have shown that *Chitinophagaceae* play a crucial role in lignin degradation, polysaccharide decomposition, and other processes [26,27]. The relative abundance of *Chitinophagaceae* in the soil decreased over time after the addition of DMPAI, thereby impacting the degradation of plant residues in the lake–terrestrial ecotone. Similarly, the relative abundance of *Halieaceae* at 10 d showed a significant decrease of 76.97% compared to the initial value at 0 d. Studies have indicated that *Halieaceae* play a significant role in metal detoxification and carbon fixation-related functions [28].

**Figure 6:**
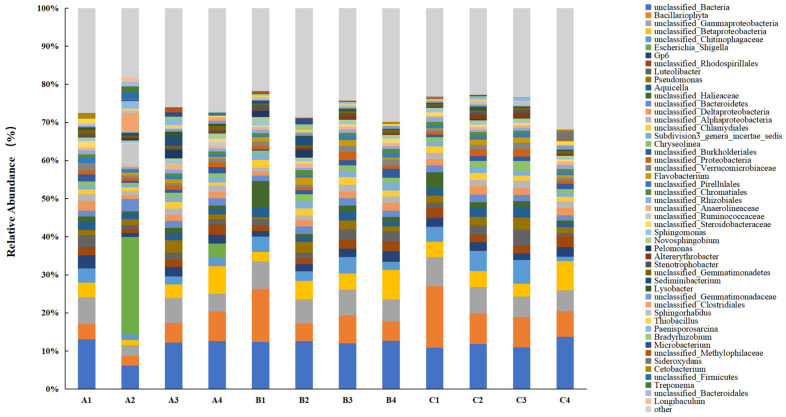
Distribution histogram of the main genera of soil bacteria in the lake–terrestrial ecotone.

The bacterial community classification at the genus level for different treatments is presented in Figure 6. Before the experiment commenced, the genera *Bacillariophyta*, *Gp6*, *Luteolibacter*, and *Aquicella* dominated the soil bacterial community in the lake–terrestrial ecotone. However, the bacterial community underwent significant changes following the addition of DMPAI. At the conclusion of the experiment, genera such as *Aquicella*, *unclassified Chlamydiales*, *Sphingomonas*, and *Stenotrophobacter* exhibited a decrease in relative abundance. *Sphingomonas* is known for its ability to degrade challenging pollutants, such as polycyclic aromatic hydrocarbons (PAHs) and hexachlorohexane (HCH), and it possesses a distinct advantage in degrading these compounds [29]. Additionally, *Stenotrophobacter* is recognized for its beneficial roles, including the secretion of phytogrowth hormones, the promotion of plant growth, and a reduction in plant morbidity [30,31]. The relative abundance of *Sphingomonas* consistently decreased across all treatment groups, suggesting that the addition of DMPAI may hinder the degradation of certain persistent pollutants in the lake–terrestrial ecotone, with limited recovery over a short period. Similarly, *Aquicella* and *Chlamydiales* are known pathogens that affect both humans and animals [32]. This observation indicates that the impact of DMPAI on environmental bacteria is dual-faceted: its bacteriostatic properties may suppress the activity of pathogenic microorganisms in the lake–terrestrial ecotone.

In contrast, several dominant genera exhibited a notable increase in relative abundance following the addition of DMPAI. For instance, the mean relative abundance of unclassified *Gemmatimonadaceae* increased by 145.87% at the end of the experiment (10 d) compared to the initial time point (0 d). *Gemmatimonadaceae* are commonly involved in nitrogen fixation processes in soil [33], suggesting that DMPAI may positively influence certain nitrogen cycling processes in the soil. During the first 6 d of the experiment, the relative abundance of *Pseudomonas* and *Flavobacterium* increased substantially following the addition of DMPAI. By the end of the experiment (10 d), the relative abundance of these genera returned to levels comparable to the initial value. This observation suggests that certain bacteria, such as *Pseudomonas* and *Flavobacterium*, which possess the ability to degrade organic pollutants, become more dominant in the community after the addition of DMPAI. The effect of DMPAI does not inhibit their activity; rather, DMPAI likely serves as a carbon source, providing energy to these bacteria.

#### 3.2.3. Functional Gene Abundance in Bacteria

The experimental results (Table 2) indicated that the relative abundance of bacterial genes associated with nitrification decreased by 26.20%, 58.82%, and 69.71% in treatments A, B, and C, respectively, at the end of the experiment (10 d) compared to the beginning. This suggests that the addition of DMPAI to the environment may lead to a reduction in the nitrification capacity of soil bacteria, with higher concentrations (B and C) exhibiting more pronounced effects. Additionally, genes associated with ureolysis function also showed negative impacts, fluctuating during the initial phase of the experiment before experiencing a more substantial decline between 6 and 10 d. In contrast, nitrogen fixation functional genes were positively influenced by low concentrations of DMPAI, potentially linked to the increased activity of bacteria such as *Gemmatimonadaceae*. However, at higher concentrations (2.0 mg L^−1^), the promoting effect was lost, although no significant inhibition was observed.

The major carbon cycle functional genes included chemoheterotrophy, chloroplasts, and phototrophy. For the chemoheterotrophy functional genes, the relative abundance exhibited a continuous decreasing trend across all treatments following the addition of DMPAI, with reductions of 73.94%, 70.13%, and 87.99% in the three treatment groups, respectively, during the experimental period. This suggests that DMPAI influences the microbial degradation of organic matter in the lake–terrestrial ecotone, even at low concentrations (0.01 mg L^−1^). This phenomenon is consistent with the observed changes in bacterial community structure.

Bacterial sulfur cycle functional genes exhibited greater sensitivity to changes in the concentration of DMPAI. Following the addition of algicide, the relative abundance of sulfur cycle functional genes decreased in all three treatments. Although recovery occurred after 6 d, the average decrease in the treatment groups remained at 78.36% by the end of the experiment. The relative abundance in treatment C was lower than that in treatments A and B. This indicates that the effects of varying concentrations of DMPAI on the sulfur cycle function of bacteria were consistent, with more pronounced effects at higher concentrations.

Additionally, functional genes associated with human pathogens and intracellular parasites were detected in all treatment groups. The relative abundance of human pathogens in treatment group A peaked at day 3, increasing nearly 30-fold compared to the pre-experiment value, and remained more than 4-fold higher than the initial value (A1) on day 10 (A4). The abundance of human pathogens in treatments B and C was suppressed to a lower level after day 6. Similarly, the relative abundance of human pathogens in treatments B and C was suppressed to a lower level after day 6. This suggests that low concentrations of DMPAI may promote the activity of pathogenic microorganisms in the soil environment, whereas increasing algicide concentrations helps to inhibit the further propagation of pathogenic bacteria.

**Figure 7:**
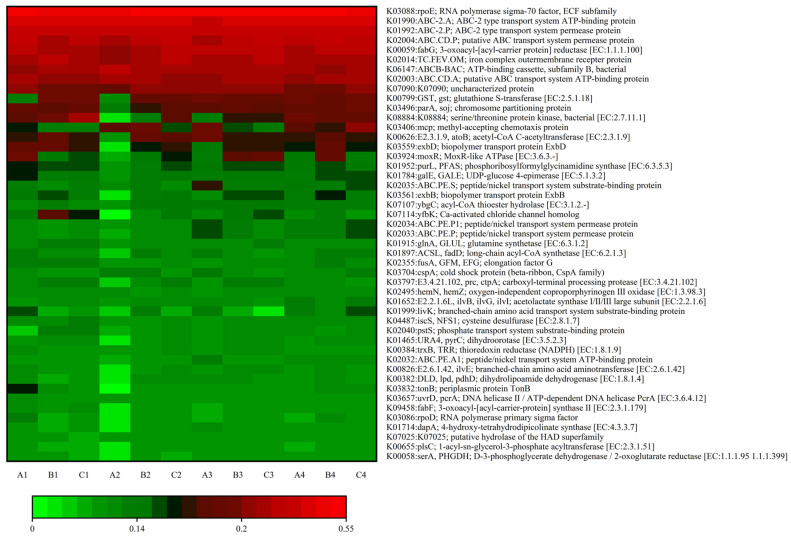
Heatmap of KO metabolic pathways in soil bacteria within the lake–terrestrial ecotone under varying concentrations of algaecide treatments.

To further elucidate the effects of varying concentrations of algicide on cellular metabolic pathways in the soil bacterial community of the lake–terrestrial ecotone, ortholog groups (KEGG orthology, KO) were used to categorize the molecular functions of genes, and the KEGG pathway database was employed to assess changes in metabolic pathways. As shown in Figure 7, the relative abundance of K08884 (serine/threonine protein kinase, bacterial [EC:2.7.11.1]), K07114 (Ca-activated chloride channel homolog), and K03406 (the methyl-tolerant chemotaxis protein) exhibited significant variations following the introduction of DMPAI into the lake–terrestrial ecotone environment. K03406 encodes a gene that assists microorganisms in mitigating the toxicity of environmental pollutants, including heavy metals. The relative abundance of K03406 showed a significant increase across treatments during the experiment, with the effect being more pronounced in treatment C, which contained a high concentration of DMPAI. Studies have shown that the K03406 gene, encoding a methyl-receptor chemotactic protein, is associated with the two-component-system metabolic pathway. This pathway, present in a wide range of Gram-negative bacteria, is essential for various bacterial life activities and represents a key mechanism through which bacteria sense environmental changes and develop corresponding regulatory strategies [34]. The increase in the relative abundance of K03406 indicates changes in the proportion of Gram-negative bacteria in the bacterial community.

Additionally, the relative abundance of the substrate-binding protein (K02035), involved in the peptide/nickel transport system increased from 3 d to 6 d in treatments A and C, while in treatment E it remained stable after a slight increase. This suggests that the addition of DMPAI enhanced the utilization of titanium and nickel in the soil by bacteria over a certain period, potentially linked to the biological detoxification mechanism. The overall upward trend in the relative abundance of peptide/nickel transport system permease proteins (K02033, K02034) throughout the experimental period further supports this observation. K00626 is a gene associated with carbon metabolism, primarily involved in the ethylmalonyl pathway, hydroxybutyrate–dicarboxylate cycle, and hydroxypropionate–hydroxybutyrate cycle. In the experiment, among the three treatment groups the relative abundance of the K00626 functional gene fluctuated the most in treatment GG, while treatment V exhibited the least change. This suggests that the K00626 functional gene may be more regulated by low concentrations of DMPAI.

## 4. Discussion

Most QACs are toxic and can cause significant toxic effects on microorganisms and other aquatic organisms even at low concentrations [35,36]. Carbajo et al. [37] conducted a series of toxicological assessments on BACs (benzalkonium chlorides, a type of QAC) in aquatic organisms and found that BACs exhibited substantial toxicity, with an EC_50_ of less than 1 mg L^−1^ in at least one assay. Due to their positive surface charge, QACs readily bind to bacterial cell membranes, thereby exhibiting broad-spectrum bactericidal properties. Studies by Agathokleous et al. [17] have shown that when mixtures of QACs are present in the environment at low concentrations, hormetic effects may occur, potentially facilitating the spread of pathogenic microorganisms. Another concern regarding QACs is that because some microorganisms harbor antibiotic resistant genes co-located with QAC resistant genes within the same integron, the introduction of large quantities of QACs into aquatic environments may promote the proliferation of drug-resistant bacteria, thereby increasing environmental risk [38].

Typically, QACs in water can be effectively removed by microorganisms in activated sludge under aerobic conditions (removal rate > 90%) due to their strong adsorption onto the sludge surface [39]. The un-degraded QACs typically remain in the residual sludge [40]. Although DMPAI is completely degraded within 36 h after entering the lake–terrestrial ecotone, its impact on microorganisms in this environment should not be overlooked. Studies have shown that QACs exhibit significant inhibitory or toxic effects on certain bacteria at low concentrations (0.021–3.9 mg L^−1^) [41,42]. As DMPAI disrupted the structure of the soil bacterial community, it led to the decline of the original dominant species, thereby promoting the growth of a variety of new species, which contributed to the increased uniformity of the bacterial community. Any low concentration (0.01 mg L^−1^) of DMPAI leads to a significant reduction in bacterial species in the soil over a short period of time; however, recovery occurs by day 10, and it does not result in long-term effects on the bacterial community. Appropriately increasing the concentration of DMPAI (e.g., 0.5 mg L^−1^) favored an increase in bacterial species in the soil. This may be because DMPAI can serve as a carbon source to provide energy for microbial life activities. At an appropriate concentration, inhibiting the activity of certain microorganisms can simultaneously promote the proliferation of other heterotrophic microorganisms. This phenomenon has also been observed in studies on other organic fungicides [43]. The results of the soil bacterial community composition analysis in the lake–terrestrial ecotone support this inference. Bacteria with DMPAI tolerance and degradation ability, such as *Pseudomonas* and *Flavobacterium*, proliferated in large numbers at the beginning of the experiment and dominated the community. As the DMPAI concentration decreased, these microorganisms rapidly declined after day 6, leading to continuous changes in the microbial community structure. During this process, Gram-negative bacteria generally exhibited stronger pollutant tolerance mechanisms, such as efflux pumping systems and lipopolysaccharide outer membrane barriers [44,45,46]. The addition of DMPAI led to a rapid increase in the proportion of Gram-negative bacteria in the community. This explains the increase in the relative abundance of the K03406 metabolic pathway. Only after DMPAI was degraded did the proportion of Gram-negative bacteria in the environment gradually recover.

The results indicated that functional genes involved in the carbon, nitrogen, and sulfur cycles were all adversely affected in the short term in the lake–terrestrial ecotone environment following the addition of DMPAI. For instance, in the nitrogen cycle, nitrification and nitrogen fixation were inhibited, and DMPAI exhibited similar effects on soil bacteria associated with the carbon and sulfur cycles. Simultaneously, the activity of certain pathogenic bacteria (e.g., *Aquicella*) was reduced due to the bacteriostatic properties of DMPAI. However, the addition of DMPAI disrupted the stability of the bacterial community. During the recovery of the community, pathogenic bacteria may proliferate over a short period, posing a risk to human and animal health. This phenomenon is more pronounced in environments with higher concentrations of DMPAI. Therefore, controlling the concentration of DMPAI used is essential. DMPAI at a concentration of 0.5 mg L^−1^ is effective in eliminating cyanobacteria in water bodies [14] and causes minimal environmental impact.

Taken together, the species composition and relative abundance of the pre-existing bacterial community can be altered in a short period after DMPAI enters the soil environment of the lake–terrestrial ecotone. However, DMPAI did not cause long-term effects on soil bacteria in the lake–terrestrial ecotone at concentrations ranging from 0.01 to 2.0 mg L^−1^. After the stress of DMPAI was alleviated, the soil bacterial communities recovered within 10 days to a structure similar to that prior to the experiment.

## 5. Conclusions

In this study, a large-scale indoor simulation device was used to carry out experiments to investigate a series of effects caused by different concentrations of DMPAI on microbial communities and their functional genes in the lake–terrestrial ecotone. However, due to the limitations of the experimental conditions, the findings still need to be further verified in a real lake environment.

(1) DMPAI at concentrations less than 2 mg L^−1^ is rapidly degraded by microorganisms in the lake–terrestrial ecotone and completely disappears in 36 h.
(2) The addition of DMPAI altered the microbial community structure in the lake–terrestrial ecotone, as evidenced by an increase in the diversity index and the proliferation of microorganisms capable of tolerating and degrading DMPAI, such as Pseudomonas and Flavobacterium, within a short period. These changes were typically observed after 10 d and generally recovered, not persisting for extended periods.
(3) Functional genes involved in carbon, nitrogen, and sulfur cycling are more impaired in a lake–terrestrial ecotone after DMPAI addition. The destabilization of the microbial community may lead to a short-term increase in pathogenic bacteria during the recovery process. This phenomenon was more pronounced in environments with higher concentrations of DMPAI. Therefore, the concentration of DMPAI should be controlled within the range of 0.5 to 2.0 mg L^−1^.

## Figures and Tables

**Table 1:** Soil chemistry in a simulation pool.

Treatment	PH	TN (‰)	TP (‰)	SOM (‰)
A	8.75	0.46	0.78	5.58
B	8.80	0.35	0.77	5.31
C	8.88	0.31	0.73	5.03

**Table 2:** Prediction of functional genes in bacterial communities of the lake–terrestrial ecotone using the FAPROTAX database.

Functional Genes		A1	B1	C1	A2	B2	C2	A3	B3	C3	A4	B4	C4
Nitrogen cycle	Aerobic nitrite oxidation	351	343	1049	36	270	179	134	142	67	152	137	239
	Nitrification	397	357	1086	51	306	315	139	157	92	293	147	329
	Nitrogen fixation	178	275	354	131	852	218	478	154	59	300	173	271
	Nitrate respiration	101	195	144	53	267	130	103	135	57	302	179	240
	Nitrate reduction	279	267	466	9645	430	187	205	212	72	1483	290	278
	Nitrogen respiration	101	195	144	53	279	130	103	135	57	302	179	240
	Ureolysis	382	100	228	60	267	231	324	104	103	103	48	68
Carbon cycle	Methanotrophy	165	26	100	3	19	3	27	19	5	13	15	32
	Methanogenesis	11	0	12	37	45	2	6	0	0	16	0	9
	Methanol oxidation	63	410	430	24	108	62	658	52	21	71	66	108
	Methylotrophy	228	436	530	46	127	65	685	71	26	91	81	140
	Chitinolysis	1207	74	465	144	204	368	597	254	102	205	84	75
	Aromatic compound degradation	52	139	43	47	142	54	148	5	0	19	1	3
	Hydrocarbon degradation	165	32	100	3	19	3	27	19	5	13	15	32
	Chloroplasts	6903	8815	23,691	1075	2393	4309	2608	3667	2613	2597	1565	1837
	Chemoheterotrophy	21,276	7079	12,891	13,907	7614	7437	8470	7073	5295	4728	3203	2546
Sulfur cycle	Sulfate respiration	220	519	95	16	31	17	51	23	6	110	104	22
	Sulfur respiration	310	49	94	15	44	25	39	12	2	8	13	26
	Sulfite respiration	5	123	19	0	21	4	0	4	1	1	2	1
	Thiosulfate respiration	0	2	0	4	22	10	19	0	0	3	0	0
	Respiration of sulfur compounds	530	568	189	31	75	42	90	35	8	118	117	48
	Dark oxidation of sulfur compounds	12	0	18	0	6	0	5	3	4	5	1	0
Others	Human pathogens: all	336	179	158	9628	208	111	150	36	20	1213	37	25
	Dark hydrogen oxidation	56	25	55	33	41	36	58	42	12	72	43	58
	Iron respiration	341	116	96	13	43	16	27	13	3	9	19	45

## Data Availability

The original contributions presented in this study are included in the article. Further inquiries can be directed to the corresponding authors.
